# Hypoxia–Induced Cytotoxic Drug Resistance in Osteosarcoma Is Independent of HIF-1Alpha

**DOI:** 10.1371/journal.pone.0065304

**Published:** 2013-06-13

**Authors:** Jennifer Adamski, Andrew Price, Caroline Dive, Guy Makin

**Affiliations:** 1 Clinical and Experimental Pharmacology, Paterson Institute for Cancer Research, Manchester, United Kingdom; 2 Institute of Cancer Sciences, Manchester Cancer Research Centre, Manchester Academic Health Sciences Centre, University of Manchester, Manchester, United Kingdom; 3 Department of Paediatric Oncology, Royal Manchester Children’s Hospital, Manchester, United Kingdom; Faculté de médecine de Nantes, France

## Abstract

Survival rates from childhood cancer have improved dramatically in the last 40 years, such that over 80% of children are now cured. However in certain subgroups, including metastatic osteosarcoma, survival has remained stubbornly poor, despite dose intensive multi-agent chemotherapy regimens, and new therapeutic approaches are needed. Hypoxia is common in adult solid tumours and is associated with treatment resistance and poorer outcome. Hypoxia induces chemotherapy resistance in paediatric tumours including neuroblastoma, rhabdomyosarcoma and Ewing’s sarcoma, in vitro, and this drug resistance is dependent on the oxygen-regulated transcription factor hypoxia inducible factor-1 (HIF-1). In this study the effects of hypoxia on the response of the osteosarcoma cell lines 791T, HOS and U2OS to the clinically relevant cytotoxics cisplatin, doxorubicin and etoposide were evaluated. Significant hypoxia-induced resistance to all three agents was seen in all three cell lines and hypoxia significantly reduced drug-induced apoptosis. Hypoxia also attenuated drug-induced activation of p53 in the p53 wild-type U2OS osteosarcoma cells. Drug resistance was not induced by HIF-1α stabilisation in normoxia by cobalt chloride nor reversed by the suppression of HIF-1α in hypoxia by shRNAi, siRNA, dominant negative HIF or inhibition with the small molecule NSC-134754, strongly suggesting that hypoxia-induced drug resistance in osteosarcoma cells is independent of HIF-1α. Inhibition of the phosphoinositide 3-kinase (PI3K) pathway using the inhibitor PI-103 did not reverse hypoxia-induced drug resistance, suggesting the hypoxic activation of Akt in osteosarcoma cells does not play a significant role in hypoxia-induced drug resistance. Targeting hypoxia is an exciting prospect to improve current anti-cancer therapy and combat drug resistance. Significant hypoxia-induced drug resistance in osteosarcoma cells highlights the potential importance of hypoxia as a target to reverse drug resistance in paediatric osteosarcoma. The novel finding of HIF-1α independent drug resistance suggests however other hypoxia related targets may be more relevant in paediatric osteosarcoma.

## Introduction

Osteosarcoma is the most common primary malignancy of bone and occurs most frequently in late childhood and early adulthood. [1] The introduction of dose intensive combination chemotherapy has increased the overall survival for osteosarcoma patients to over 70%. [2], [3] However in those with metastasis and in those who relapse, prognosis remains poor with survival rates of only 20–30%. [4], [5] There has been no improvement in the survival of osteosarcoma patients in the last 20 years and therefore new therapeutic options are urgently needed.

In vitro evidence of hypoxia-induced drug resistance exists for a wide variety of cytotoxic agents in a wide variety of adult tumour types. [6]–[12] Hypoxia is able to induce resistance to etoposide and vincristine in neuroblastoma cells and doxorubicin, vincristine and actinomycin-D in rhabdomyosarcoma and Ewing’s sarcoma cells. [13], [14] Markers of hypoxia including hypoxia-inducible factor-1 (HIF-1), vascular endothelial growth factor (VEGF) and carbonic anhydrase IX (CA IX) can be detected in osteosarcomas [15]–[17] and the presence of these markers correlates with poor patient outcome, suggesting that hypoxia has an important role in osteosarcoma. [15], [17], [18] The effect of hypoxia on drug response in osteosarcoma has not been shown.

The main transcription factor responsible for the cellular adaptation to hypoxia is HIF-1. HIF-1 is comprised of 2 sub-units, a constitutionally expressed beta unit (HIF-1-β) and an oxygen regulated alpha unit (HIF-1α or HIF-2α). [19], [20] In the presence of oxygen the alpha subunits are hydroxylated by oxygen-dependant prolyl hydroxylases allowing binding to the Von Hippel Lindau (VHL) protein and targeting for ubiquitination and degradation. In hypoxia, hydroxylation does not occur and the alpha subunits stabilise, dimerise with HIF-1β and translocate to the nucleus where they regulate the transcription of over 100 target genes, many of which are directly or indirectly involved in drug resistance. [21] Known HIF-1 transcriptional targets may induce drug resistance by affecting drug transport (eg. increased p-glycoprotein [22]) or drug targets (eg. decreased topiosomerase II [23]) or by changing the response to drugs, for instance by modifying drug-induced apoptosis [8], reducing drug-induced senescence [11], or inducing autophagy in response to drugs. [24] Hypoxia-induced drug resistance is dependent on HIF-1 in the majority of cases and inhibition of HIF-1 re-sensitises cells to drug treatment in hypoxia. [6], [8]–[11], [13], [22]–[29] Thus in many tumour types HIF-1 is a valid target to reverse hypoxia-induced drug resistance.

A number of other cellular pathways are differentially regulated in hypoxia and may also contribute to hypoxia-induced drug resistance. Wild-type p53 is inactivated in some tumour cells in hypoxia, inducing resistance to p53-mediated apoptosis [30]–[33], and in some tumour types hypoxia-induced drug resistance occurs only in cell lines with wild-type p53. [25] Activation of the phosphoinositol-3-kinase (PI3K) pathway, nuclear factor kappa-B (NFκB), cycloxygenase-2 (COX-2), activator protein-1 (AP-1), c-jun, Pim-1 and STAT-3 in hypoxia have all been found to induce drug resistance, mainly by a reduction in drug-induced apoptosis. [12], [31], [34]–[39] Importantly, inhibiting this activation sensitises cells to cytotoxic agents in hypoxia, and they are thus possible targets to reverse hypoxia-induced drug resistance.

In this work we show for the first time that osteosarcoma cells are resistant to the clinically relevant cytotoxics cisplatin, doxorubicin and etoposide in hypoxia and that this resistance is not dependent on HIF-1, or on an active PI3K pathway, suggesting the need to investigate other hypoxia-related targets in this tumour type.

## Results

### Hypoxia Induces Drug Resistance in Osteosarcoma Cells and Reduces Cytotoxic-induced Apoptosis

**Figure 1:**
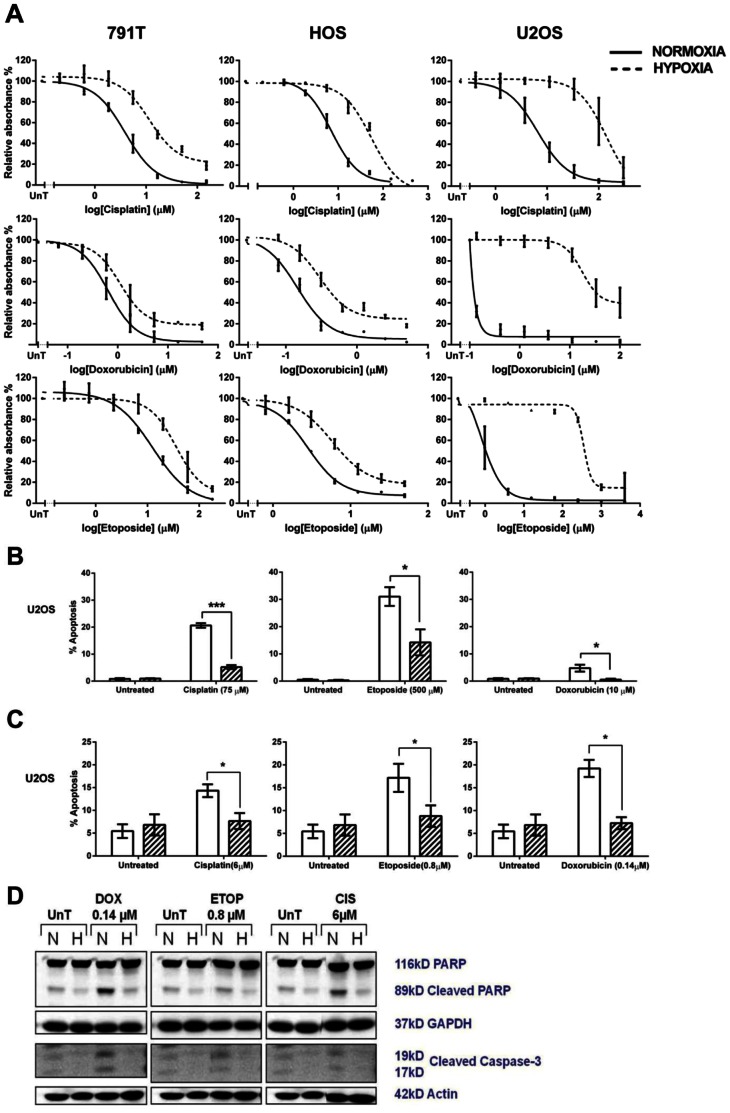
Hypoxia leads to cytotoxic drug resistance and reduces cytotoxic-induced apoptosis in osteosarcoma cells. A, Following a 24 hour pre-treatment incubation period in normoxia or hypoxia 791T, HOS and U2OS cells were treated with a range of concentrations of cisplatin (791T 0–150 µM; HOS 0–450 µM; U2OS 0–300 µM), etoposide (791T 0–180 µM; HOS 0–50 µM; U2OS 0–4000 µM) or doxorubicin (791T 0–48 µM; HOS 0–5 µM; U2OS 0–100 µM) for 1 hour. After a further 72 hours an SRB assay was performed. Graphs show the mean absorbance relative to the untreated controls (UnT) against log cisplatin, etoposide or doxorubicin concentration and are the mean ± SEM of 3 independent experiments. The difference between drug response in hypoxia and normoxia is highly significant p<0.001 in all cases (2-way ANOVA). B, 48 hours after exposure to a 1 hour pulse of doxorubicin (10 µM), etoposide (500 µM) or cisplatin (75 µM) in normoxia or hypoxia U2OS cells were stained with DAPI and morphologically apoptotic cells counted with a fluorescent microscope. Graphs represent the percentage of apoptotic cells in normoxia and hypoxia. Data are the mean ± SEM of 3 independent experiments. * indicates p<0.05 and *** indicates p<0.001 determined by the 2-tailed student t-test. C, 72 hours after exposure to a 1 hour pulse of doxorubicin (0.14 µM), etoposide (0.8 µM) or cisplatin (6 µM) in normoxia or hypoxia U2OS cells were stained with annexin V and 7-AAD and analysed by flow cytometry. Annexin V positive and/or 7-AAD positive cells were counted as apoptotic and graphs represent the percentage of apoptotic cells and are the mean ± SEM of 3 independent experiments.* indicates p<0.05 determined by the 2-tailed student t-test. D, Protein from this experiment was immunoblotted for PARP, cleaved PARP, caspase-3 and cleaved caspase-3 The amount of cleavage of PARP and caspase-3 was indicative of the amount of apoptosis occurring at that time point and was compared between normoxia and hypoxia.

In all three osteosarcoma cell lines 24 hr exposure to 1% oxygen, followed by 1 hr exposure to cytotoxic agent, and then followed by a further 72 hrs exposure to 1% oxygen (hereafter referred to as hypoxia), lead to significant resistance to cisplatin, doxorubicin and etoposide (p<0.01 to p<0.001 2-way ANOVA) in an SRB assay (Figure 1A). Returning the cells to 21% oxygen after cytotoxic exposure did not induce drug resistance (data not shown), as we have previously reported in neuroblastoma. [13] Hypoxia-induced drug resistance was particularly pronounced in U2OS cells, with a 439 fold increase in the IC50 for etoposide (Table 1). The pattern of hypoxia-induced resistance to etoposide and doxorubicin, with 791T cells showing the least and U2OS cells the greatest, reflected the relative sensitivity of the three osteosarcoma cell lines to these agents in normoxia; the IC50 for etoposide in 791T cells in normoxia was 12.6 µM as compared to 0.8 µM in U2OS cells, whilst for doxorubicin the values were 0.7 µM and <0.14 µM. However this was not the case with cisplatin to which all three cell lines were similarly sensitive in normoxia.

**Table 1:** IC_50_ doses for osteosarcoma cells by sulphorhodamine-B (SRB) assay.

Drug	Cell line	Normoxia		Hypoxia		Fold Change
		IC_50_ (µM)	95% confidence interval	IC_50_ (µM)	95% confidence interval	
**Cisplatin**	791T	3.90	3.14–4.84	11.56	7.94–16.83	3.0
	HOS	7.23	6.06–8.63	54.11	39.33–74.44	7.5
	U2OS	5.85	3.8–9.02	97.22	41.0–174.3	16.6
**Etoposide**	791T	12.59	8.5–18.66	35.75	22.34–57.19	2.8
	HOS	2.85	2.62–3.09	5.86	4.51–7.61	2.1
	U2OS	0.8	0.32–1.99	351.5	107.9–1145.0	439.4
**Doxorubicin**	791T	0.65	0.48–0.86	1.12	0.76–1.65	1.7
	HOS	0.15	0.12–0.18	0.3	0.24–0.38	2.1
	U2OS	<0.14	N/A	17.6	9.68–32.01	>125.8

Hypoxia induces resistance to cytotoxic drugs by suppressing apoptosis. [8], [13], [14], [40] In U2OS cells there was a significant reduction in apoptosis measured by morphological changes (p<0.05–001 2-tailed student t-test) (Figure 1B) and annexin V/7-AAD positivity (p<0.05–0.01 2-tailed student t-test) (Figure 1C), and a reduction in levels of cleaved caspase-3 and PARP on western blotting (Figure 1D) in hypoxia compared to normoxia. A significant reduction in apoptosis was also observed in HOS cells exposed to all 3 drugs and in 791T cells exposed to cisplatin and doxorubicin in at least 2 out of the 3 assays (data not shown). In 791T cells there was no significant difference in etoposide-induced apoptosis between normoxia and hypoxia. 791T cells exposed to etoposide have the least significant difference in response between normoxia and hypoxia on SRB assay (p<0.01 2-way ANOVA (Figure 1A)). Thus hypoxia-induced resistance to cytotoxic agents in osteosarcoma cells is due to reduced drug-induced apoptosis.

### Hypoxia-induced Drug Resistance in Osteosarcoma Cells is not Dependent on HIF-1

**Figure 2:**
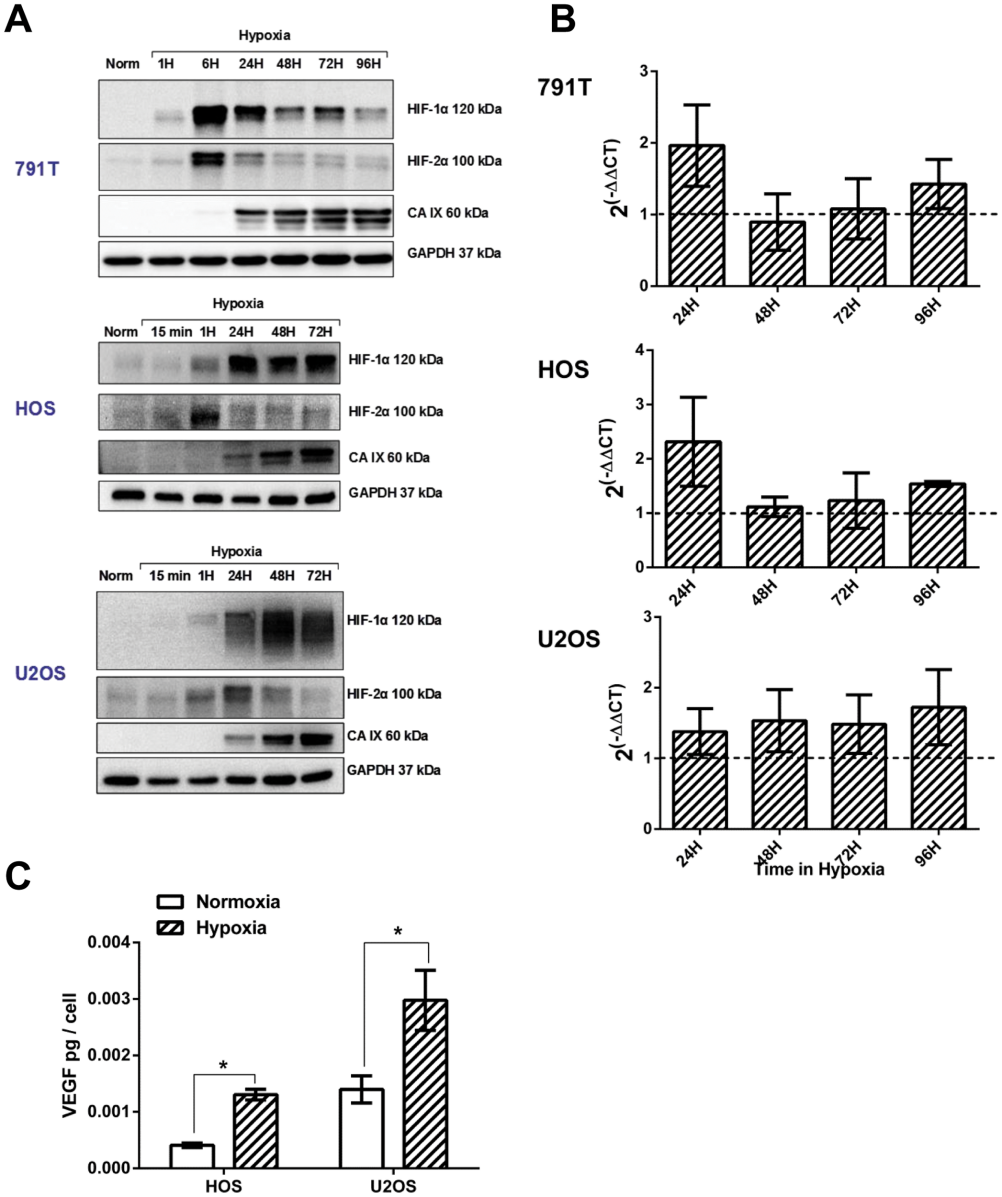
Regulation of HIF-1 expression in osteosarcoma cells. A, Western blots showing the time course of HIF-1α and HIF-2α stabilisation and protein levels of the HIF-1 transcriptional target carbonic anhydrase IX (CA IX) in 791T, HOS and U2OS cells after exposure to hypoxia. GAPDH is a loading control. Data are representative of 3 independent experiments. B, Graphs show 2^(−ΔΔCT)^ where CT is the cross-threshold and represents the change in Glut-1 mRNA expression with time in 791T, HOS and U2OS cells in hypoxia relative to normoxia, where 1 would be equivalent expression in normoxia and hypoxia and greater than 1 represents an increase in hypoxia relative to normoxia. Data are the mean ± SEM of 2 independent experiments. C, VEGF-A levels in the supernatant of HOS and U2OS cells detected by enzyme-linked immunosorbent assay after 24 hours in normoxia or hypoxia, normalised to cell number. Data are the mean ± SEM of 3 independent experiments. * indicates p<0.05 as determined by the 2-tailed student t-test.

**Figure 3:**
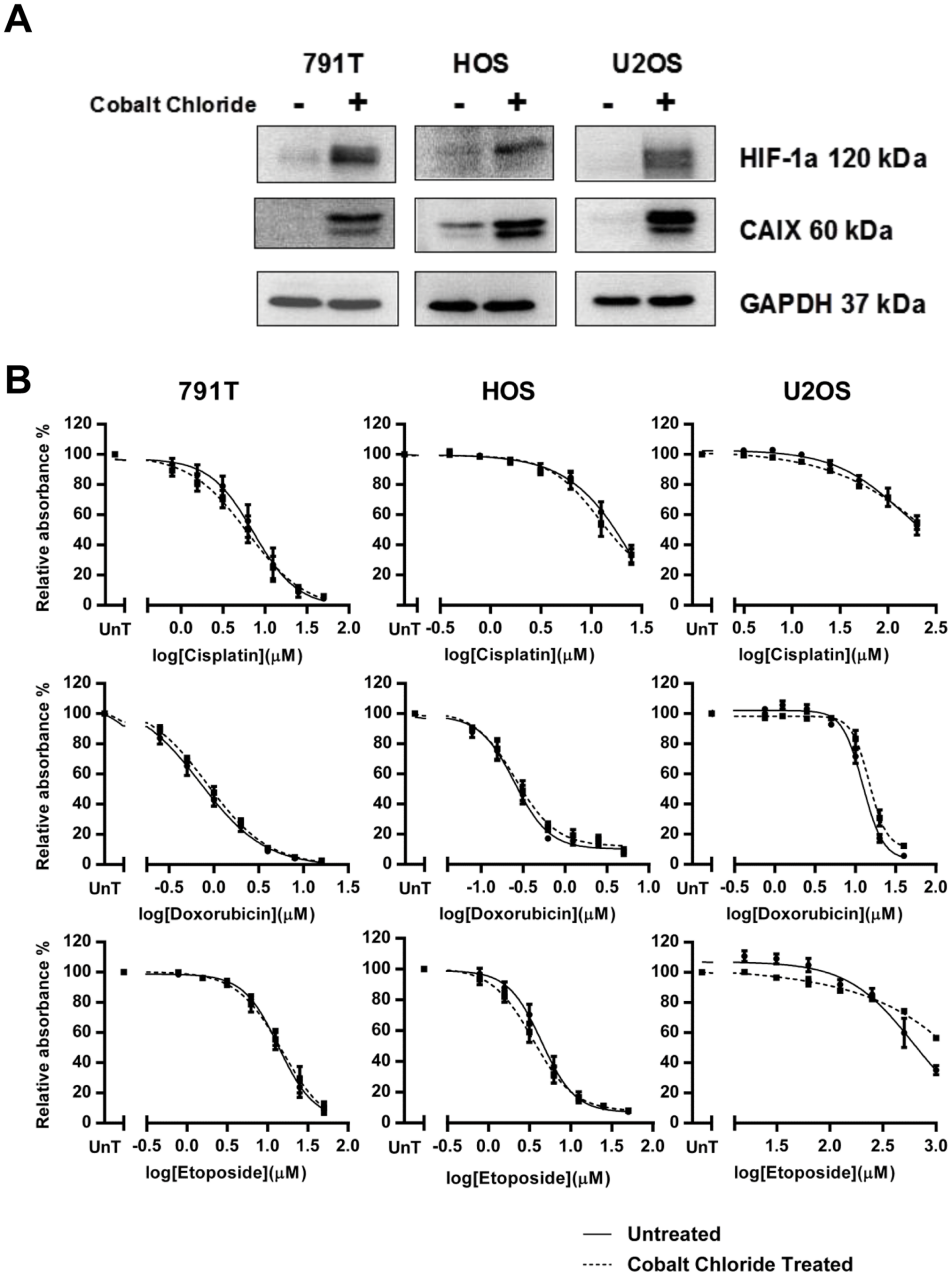
Significant hypoxia-induced resistance remains despite inhibition of HIF-1 by shRNAi or siRNA. A, Stable HOS clones expressing shRNAi to HIF-1α (C5) and firefly luciferase as a control (L4) were incubated in normoxia or hypoxia for 24 hours before exposure to cisplatin (0–150 µM), doxorubicin (0–2.5 µM) or etoposide (0–50 µM) for 1 hour. An SRB assay was performed 72 hours after treatment. B, Western blotting performed on cell lysates from cells simultaneously maintained in hypoxia shows reduced expression of HIF-1α and CA IX, indicating suppressed transcriptional activity, throughout the experiment. D, Stable 791T clones expressing shRNAi to HIF-1α (C24) and firefly luciferase as a control (L3) were similarly processed and treated with doxorubicin (0–48 µM) or etoposide (0–180 µM) for 1 hour. C, Whole cell lysates from cells simultaneously plated were harvested at 24 hours (24H) (at treatment) and 96 hours (96 H) of hypoxia (the experiment end). Western blotting for HIF-1α and CA IX shows suppression of HIF-1α expression and transcriptional activity. E, 791T cells were transiently transfected with siRNA to HIF-1α or a non-targeting control (NT). 8 hours after transfection the hypoxic arm was transferred to hypoxia and after 16 hours cells were treated with cisplatin for 1 hour (0–150 µM). 72 hours after treatment cells were assessed by SRB assay. F, Western blotting on cell lysates collected from cells simultaneously transfected after 24 hours (24 H) and 96 hours (96 H) of hypoxia shows suppression of HIF-1α and CA IX expression. All graphs show the mean absorbance relative to the untreated controls against log drug concentration and are the mean of 3 independent experiments ± SEM. The difference in the drug response of the shRNAi clones and the siRNA transfected cells between hypoxia and normoxia remains highly significant in all cases despite HIF-1α suppression (p<0.001, 2-way ANOVA). Western blots are representative of 3 independent experiments. GAPDH and actin were loading controls.

**Figure 4:**
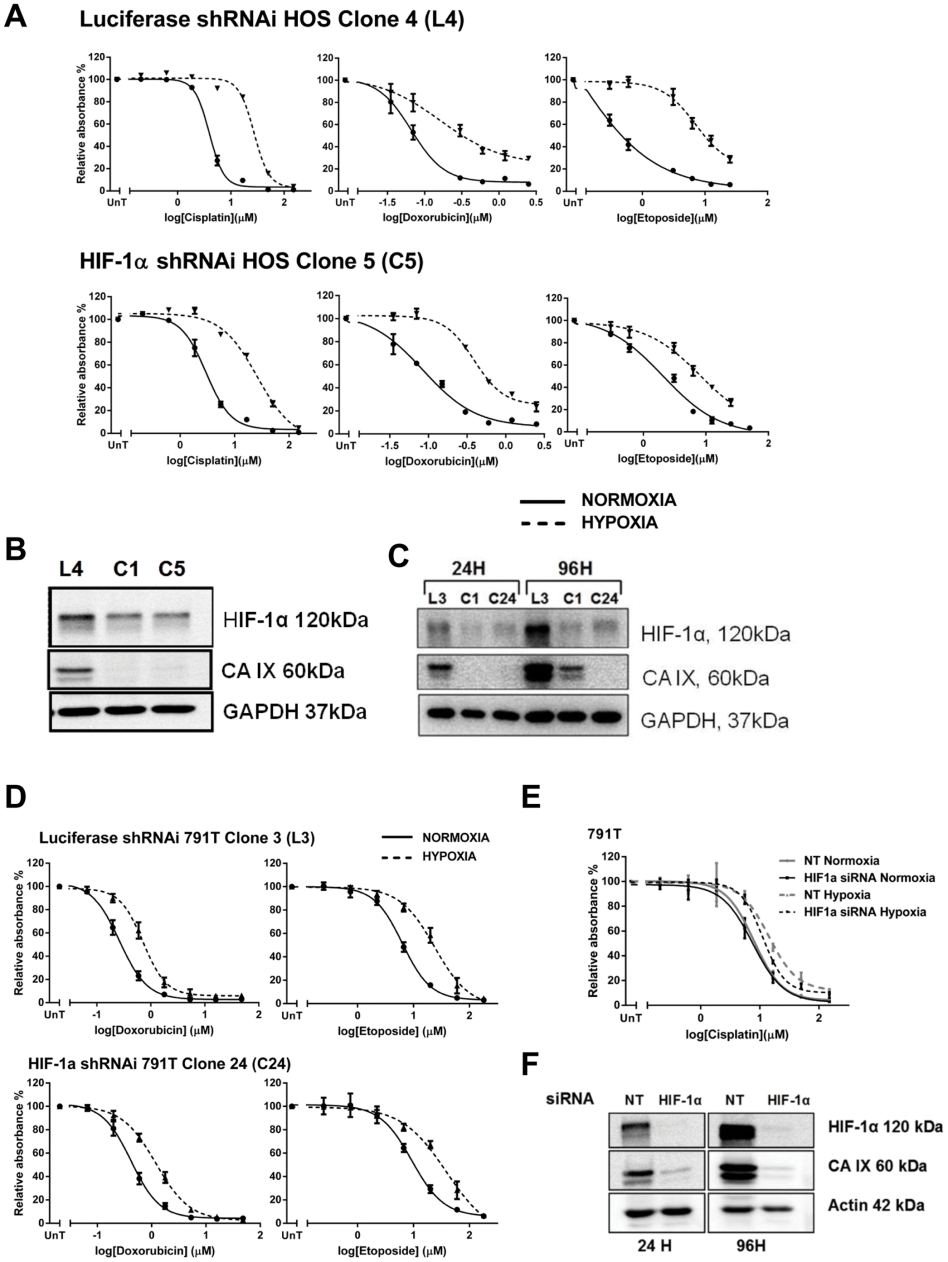
Osteosarcoma cells expressing dominant-negative HIF-1α remain resistant to cisplatin, doxorubicin and etoposide in hypoxia. U2OS cells were transiently transfected with the pEF-IRES-P-HIF-no-TAD-EGFP vector (Dominant-negative HIF) (DN) or the empty vector control (EV). Following a 24 hour pre-treatment incubation period in either normoxia (N) or hypoxia (H) cells were exposed to a range of concentrations of cisplatin (0–300 µM), doxorubicin (0–100 µM) or etoposide (0–4000 µM) for 1 hour. 72 hours after treatment cells were fixed and assessed by SRB assay (B). Simultaneously transfected and plated cells were maintained in normoxia or hypoxia and harvested at 24 hours hypoxia (at time of treatment) or 96 hours hypoxia (at the end of the experiment). RNA was extracted and qPCR performed for CA IX and Glut-1 expression (A). Graphs show 2^(−ΔΔCT)^ where CT is the Cross Threshold and represents the change in mRNA expression in hypoxia relative to normoxia, where 1 would be equivalent expression in normoxia and hypoxia and greater than 1 represents an increase in hypoxia relative to normoxia. Data are the mean ± SEM of 3 independent experiments. * indicates p<0.05 as determined by the 2-tailed student t-test.

**Figure 5:**
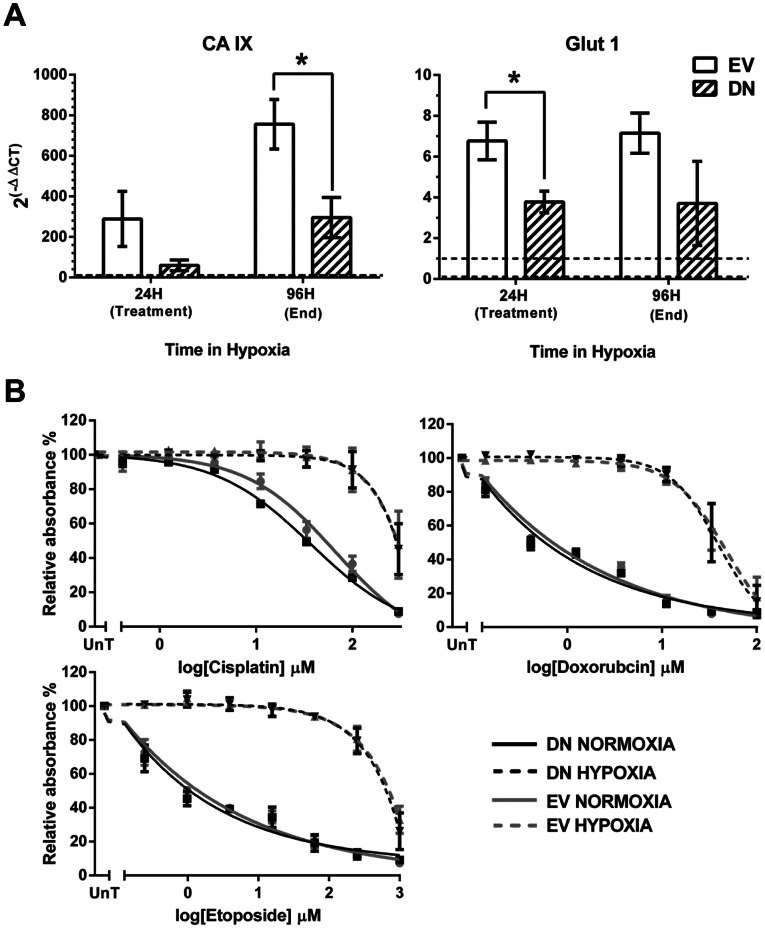
Osteosarcoma cells treated with the small molecule inhibitor of HIF-1α NSC134754 remain resistant in hypoxia. A, U2OS cells were treated with 20 µM NSC-134754 for 24 hours in hypoxia prior to exposure to a range of concentrations of cisplatin (0–300 µM), doxorubicin (0–100 µM) or etoposide (0–4000 µM) for 1 hour. Untreated controls were exposed to the same concentration ranges of cisplatin, doxorubicin and etoposide in normoxia and hypoxia. 72 hours after treatment cells were fixed and a SRB assay performed Graphs show the mean absorbance relative to the untreated controls (no chemotherapy agent) and are the average of 3 independent experiments ± SEM. B, Simultaneously plated cells treated with NSC134754 and incubated in hypoxia for 24 hours (time of treatment) or 96 hours (end of experiment) were harvested for whole cell lysates and western blotting performed for HIF-1α and CA IX. Western blots are representative 3 independent experiments with GAPDH used as a loading control. The difference between the response to cytotoxics in normoxia and hypoxia remains highly significant despite treatment with NSC134754 (p<0.001 2-way ANOVA).

**Figure 6:**
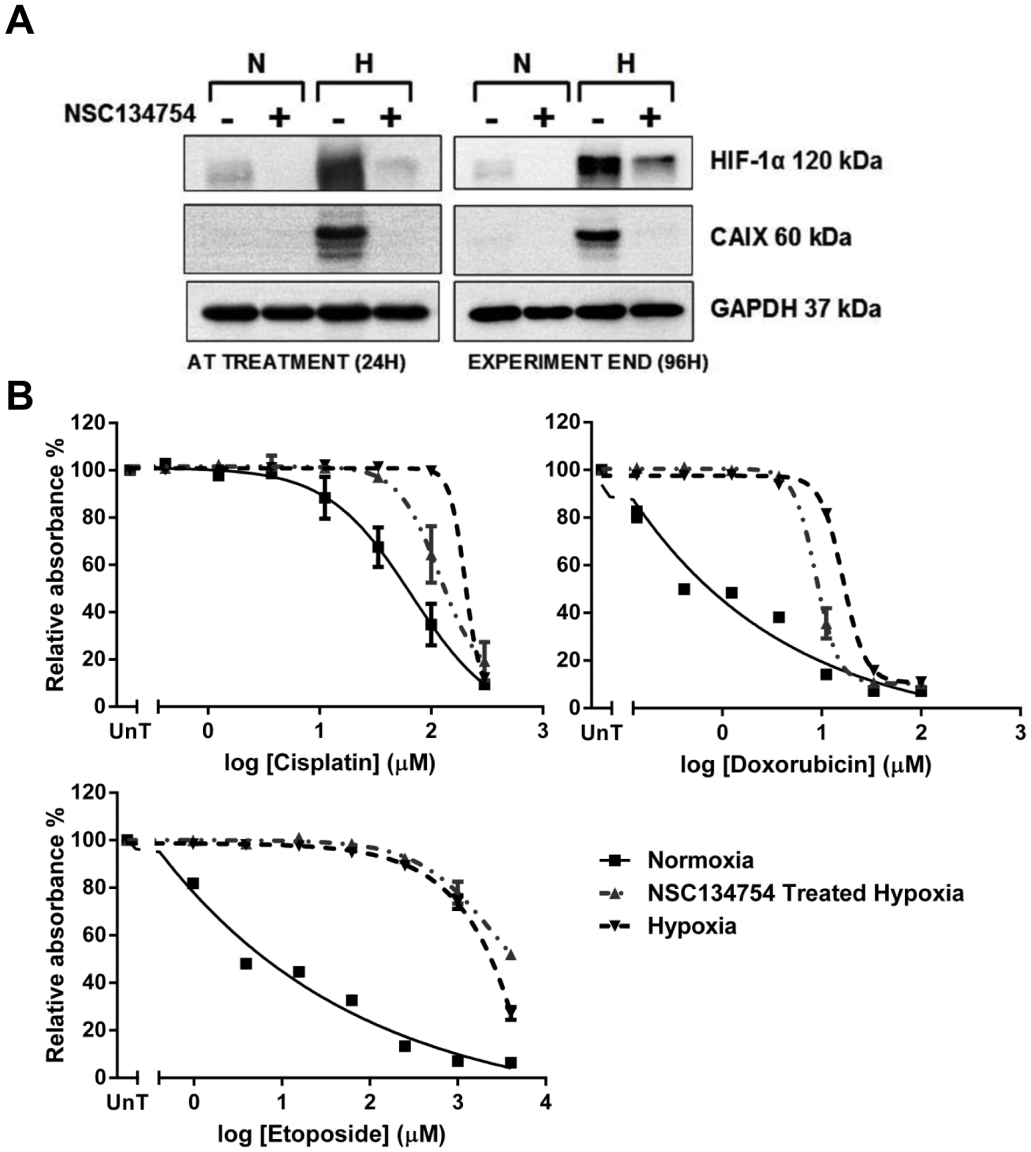
Cobalt Chloride stabilises and transcriptionally activates HIF-1α in normoxia but does not induce resistance. B, 24 hours after plating osteosarcoma cells were treated with cobalt chloride (791T 50 µM; HOS 25 µM; U2OS 25 µM) for 24 hours before treatment with a range of concentrations of cisplatin (791T 0–50 µM; HOS 0–25 µM; U2OS 0–200 µM), doxorubicin (791T 0–16 µM; HOS 0–5 µM; U2OS 0–40 µM) or etoposide (791T 0–50 µM; HOS 0–50 µM; U2OS 0–1000 µM). Following a one hour drug exposure cells were incubated with or without cobalt chloride for a further 72 hours before fixing and performing a sulphorhodamine-B assay. Graphs show the mean absorbance relative to the untreated controls (UnT) against the log of the drug concentrations and are the average of 3 independent experiments ± SEM. A, Whole cell lysates of cells treated with the above doses of cobalt chloride for the length of the experiment (96 hours) were harvested for western blotting to determine HIF-1α stabilisation and expression of downstream target CA IX. The western blots are representative of 3 independent experiments with GAPDH as a loading control.

Hypoxia-induced drug resistance is usually dependent on HIF-1. Hypoxia rapidly stabilised HIF-1α and HIF-2α in all three osteosarcoma cell lines and the corresponding up-regulation of CA IX protein levels (Figure 2A), Glut-1 mRNA levels (Figure 2B) and levels of secreted VEGF-A (Figure 2C), indicates that it is transcriptionally active in these cell lines. HIF-1 was stabilised in normoxia by exposing cells to cobalt chloride at either 50 µM (791T) or 25 µM (HOS and U2OS) for 24 hrs. Functional activity of cobalt chloride stabilised HIF-1 was confirmed by an increase in protein levels of CA IX (Figure 3A). Despite this activation of the HIF-1 pathway in normoxia, 24 hr cobalt chloride treatment did not induce drug resistance (Figure 3B), suggesting that transcriptionally active HIF-1 is not sufficient for hypoxia-induced drug resistance in osteosarcoma cells. To further investigate the role of HIF-1 in hypoxia-induced drug resistance in HOS and 791T cells, stable clones were generated in which HIF-1α was suppressed by short-hairpin RNA interference (shRNAi). Significant resistance to cisplatin, doxorubicin and etoposide remained in hypoxia compared to normoxia (Figure 4A), despite a reduction in HIF-1α protein levels sufficient to prevent transcription of CA IX (Figure 4B), and there was no significant difference in hypoxia-induced resistance to cisplatin, etoposide and doxorubicin between the HIF-1α and the luciferase repressed cells, in which HIF-1α levels and function were normal (Figure 4A, 4B). Similarly 791T HIF-1α shRNAi cells remained significantly resistant to doxorubicin and etoposide in hypoxia compared to the luciferase shRNAi control (Figure 4D), despite significantly reduced HIF-1α protein levels and suppressed HIF-1 function (Figure 4C). Both these results were verified in second HIF-1α suppressed clones (data not shown). In 791T cells highly significant (p<0.001, 2-way ANOVA) resistance to cisplatin in hypoxia remains after transient transfection of HIF-1α short interfering RNA (siRNA) (Figure 4E), despite reduction in protein levels of HIF-1α and CA IX (Figure 4F). Thus in HOS and 791T cells, suppression of HIF-1α sufficient to inhibit the transcriptional activity of HIF-1 does not prevent hypoxia from inducing significant resistance to cisplatin, doxorubicin and etoposide. HIF-1 function in U2OS cells was inhibited by transient transfection of a dominant negative HIF vector (DN-HIF) expressing a truncated HIF-1α which lacks the trans-activation domain. [6] Despite functional inhibition of HIF-1 (Figure 5A), significant resistance to cisplatin, doxorubicin and etoposide remained in hypoxia with no observable difference in drug response between the DN transfected cells and the empty vector controls (Figure 5B). Finally the small molecule NSC134754, an inhibitor of both HIF-1α and HIF-2α, was used. [41] NSC134754 reduced HIF-1α protein levels in U2OS cells in hypoxia, and reduced levels of CA IX (Figure 6A). Significant hypoxia-induced resistance to cisplatin, doxorubicin and etoposide remained despite this functional inhibition of HIF-1 (Figure 6B). The failure of HIF-1 inhibition, by a range of methods, to significantly impact on the resistance to cisplatin, doxorubicin and etoposide induced by hypoxia in any of the 3 osteosarcoma cells, suggests strongly that hypoxia-induced drug resistance is not dependent on HIF-1 in these cells.

### Hypoxia Activates the PI3K/Akt Pathway in Osteosarcoma Cells However Inhibition of this Pathway does not Affect Hypoxia-induced Drug Resistance

**Figure 7:**
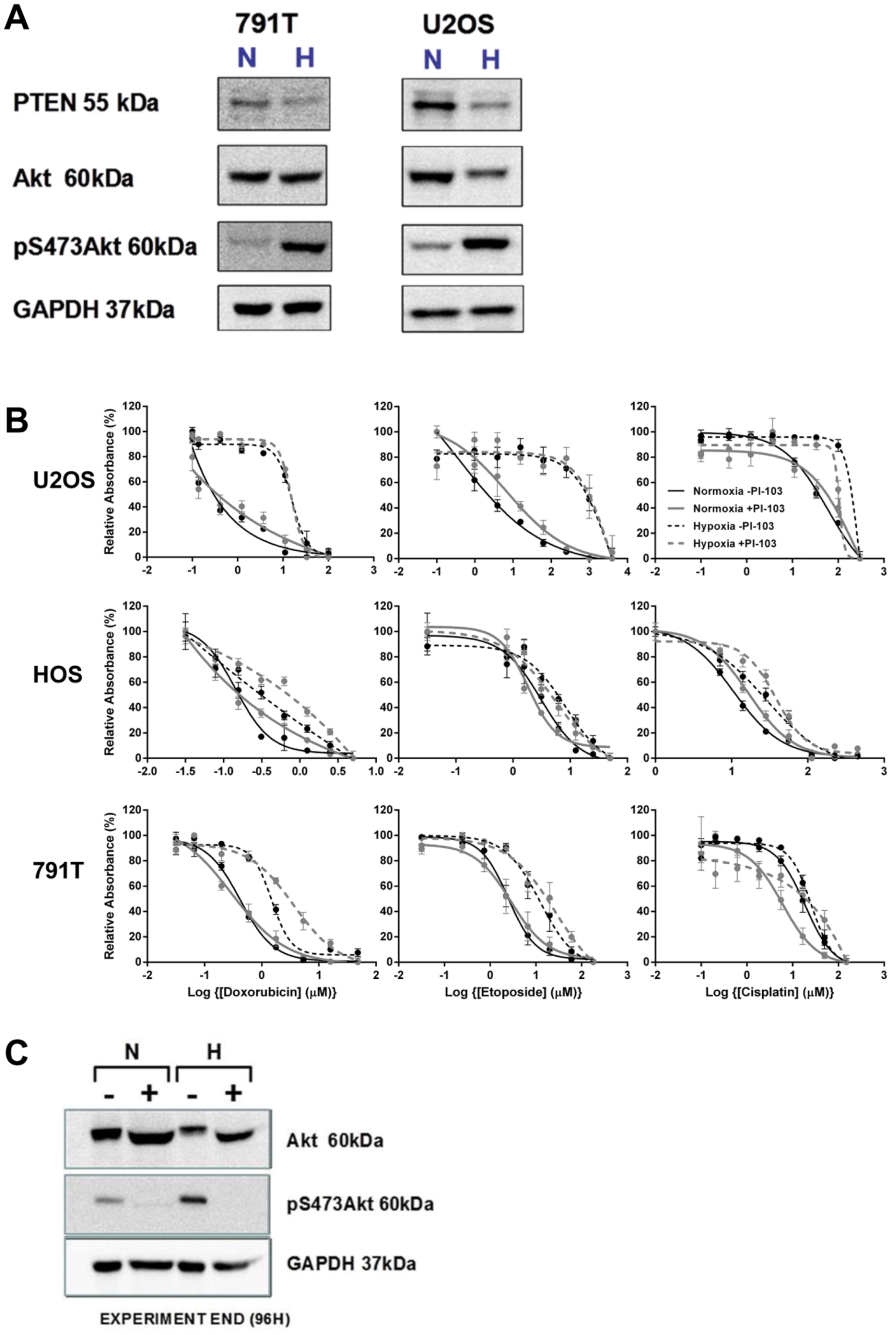
Akt is activated in hypoxia in osteosarcoma cells however hypoxia-induced resistance remains despite PI3K inhibition. A, 791T, HOS and U2OS osteosarcoma cells were incubated in normoxia (N) or hypoxia (H) for 48 hours before lysates were analysed for PTEN, Akt and Akt phosphorylated at serine 473 by western blotting. Phosphorylation and therefore activation of Akt is seen in hypoxia in 791T and U2OS cells, coinciding with a reduction of PTEN. B, to determine the effect of PI3K inhibition on drug sensitivity U2OS cells were exposure to a range of concentrations of cisplatin (0–300 µM), doxorubicin (0–100 µM) or etoposide (0–3000 µM) immediately following treatment with or without 1 µM PI-103 in normoxia and hypoxia. After 72 hours in an SRB assay was performed. The concentration of 1 µM PI-103 was maintained throughout. C, A western blot analysis for Akt and phosphorylated Akt performed after 24 hours hypoxia, at the time of treatment with doxorubicin, and 96 hours hypoxia, at the end of the experiment, showed inhibition of Akt phosphorylation. Despite inhibition of Akt activation, there remained a significant difference in response to cisplatin, doxorubicin and etoposide between normoxia and hypoxia (p<0.0001, 2-way ANOVA). The graph represents the mean relative absorbance of 3 independent experiments ± SEM. Results are normalised so that cells treated with the highest concentration of doxorubicin represent 0% and untreated cells (no doxorubicin) represent 100% in each case. Western blot analyses are representative of 3 independent experiments with GAPDH as a loading control.

Activation of PI3K and Akt prevents apoptosis and induces drug resistance in both normoxia and hypoxia and PI3K inhibition is able to reverse this resistance. [12], [42]–[44] In both U2OS and 791T cells hypoxia increased protein levels of pS473 Akt, indicating activation of the PI3K pathway in these cells in hypoxia (Figure 7A). In U2OS and 791T cells protein levels of PTEN, a negative regulator of PI3K, were reduced in hypoxia. HOS cells have an aberrantly activated PI3K pathway with strong expression of PTEN, Akt and pS473 Akt in both normoxia and hypoxia (data not shown). However, despite inhibition of PI3K activation by the small molecule inhibitor PI-103, shown by reduced pS473 Akt levels (Figure 7B), significant hypoxia-induced drug resistance remains, regardless of the scheduling of PI3K inhibition relative to cytotoxic exposure (Figure 7C).

### Phosphorylation of p53 at Serine 15 in Response to Drug Exposure is Reduced in Hypoxia in Osteosarcoma Cells

**Figure 8:**
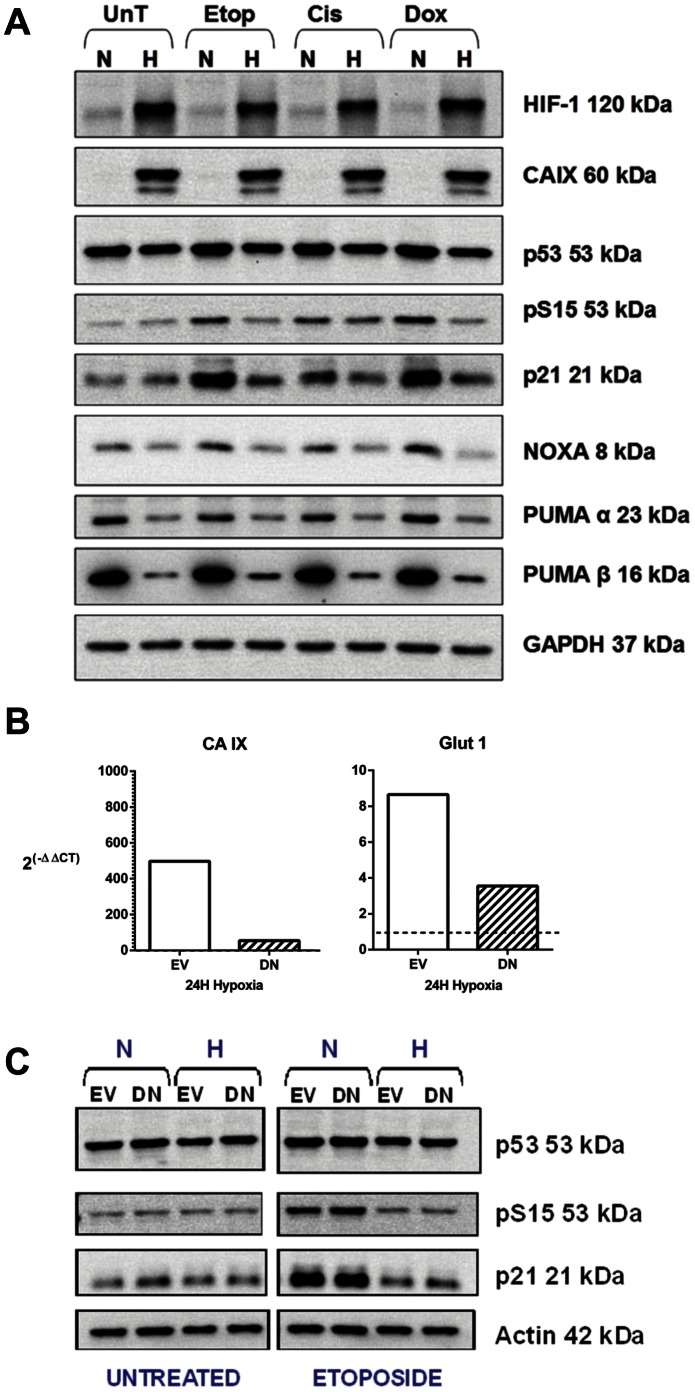
Hypoxia reduces DNA damage-induced p53 phosphorylation at serine 15, irrespective of HIF-1 inactivation. A, 24 hours after exposure to 1 µM etoposide, 6 µM cisplatin or 0.14 µM doxorubicin for 1 hour in normoxia (N) or hypoxia (H) cells were harvested and western blotting performed for p53, p53 phosphorylated at serine 15, indicative of DNA damage, and the downstream targets of p53, p21, PUMA and NOXA. Treated samples were compared to untreated controls (UnT). GAPDH was a loading control. Data are representative of 3 independent experiments. A reduction of p53 phosphorylation at serine 15 was seen in hypoxia compared to normoxia following exposure to the DNA damaging agents. B, U2OS cells were transiently transfected with the pEF-IRES-P-HIF-no-TAD-EGFP vector (Dominant-negative HIF) (DN) or the empty vector control (EV). After a 24 hour pre-treatment incubation period in either normoxia (N) or hypoxia (H) cells were exposed to 1 µM etoposide and incubated for a further 24 hours before whole cell extracts harvested and western blotting performed for p53, p53 phosphorylated at serine 15, indicative of DNA damage, and p53 transcriptional target p21. Etoposide treated samples were compared to untreated controls. Actin was a loading control. Data are representative of 2 independent experiments. C, Simultaneously transfected cells were maintained in normoxia or hypoxia and harvested at 24 hours hypoxia, the time of treatment. RNA was extracted and qPCR performed for CA IX and Glut-1 expression. Graphs show 2^(−ΔΔCT)^ where CT is the Cross Threshold and represent the change in mRNA expression in hypoxia relative to normoxia, where 1 would be equivalent expression in normoxia and hypoxia and greater than 1 represents an increase in hypoxia relative to normoxia. Data show mRNA expression from cells lysed in (B) and are representative of 2 independent experiments. Reduced phosphorylation of p53 at serine 15 and p21 protein levels were seen in hypoxia following etoposide treatment despite the inhibition of HIF-1 transcriptional activity.

Cisplatin, etoposide and doxorubicin exert their cytotoxic effect through the activation of p53 and the initiation of apoptosis. [45], [46] Oncogenically transformed cells undergo p53 dependent apoptosis in hypoxia, therefore hypoxia selects for cells which are deficient in p53. [47] In p53 wild type cells, suppression of p53 activity protects against cytotoxic-induced apoptosis. [30], [32], [33], [48] The inactivation of p53 in hypoxia is both HIF-1 dependent [33], [49], and HIF-1 independent. [48] In p53 wild type U2OS cells p53 protein was readily detectable in untreated cells, suggesting protein stabilisation. However phosphorylation of p53 protein on serine 15, an indication of p53 activation, was only detected after exposure to cytotoxic drugs (Figure 8A). Phosphorylation of p53 on serine 15 after exposure to cisplatin, etoposide and doxorubicin, was reduced in hypoxia compared to normoxia (Figure 8A), correlating with reduction in the protein levels of the known p53 transcriptional targets p21 and NOXA, suggesting that this reduction in p53 phosphorylation leads to a reduction in the transcriptional activity of p53 in hypoxia. To investigate whether p53 inactivation in hypoxia was dependent upon functional HIF-1, U2OS cells were transiently transfected with the DN-HIF vector. Despite functional inhibition of HIF-1 (Figure 8B) reduction in p53 phosphorylation on serine 15 and p21 protein levels after etoposide exposure in hypoxia were not altered (Figure 8C). This suggests reduced p53 activation in hypoxia in U2OS cells is not dependent on functional HIF-1. p53 inactivation may thus be contributing to reduced cytotoxic drug-induced apoptosis in hypoxia in U2OS cells.

## Discussion

Hypoxia-induced drug-resistance has been observed in vitro in rhabdomyosarcoma, Ewing’s sarcoma and neuroblastoma, [13], [14] although this is not a universal phenomenon, and hypoxic sensitisation has also been reported. Cytotoxic drug resistance in hypoxia can vary between tumour type and with drug used. [13], [50], [51] Evidence exists of the importance of hypoxia in osteosarcoma, but the effect of hypoxia on the response of osteosarcoma cells to clinically relevant cytotoxic drugs has not been reported.

Highly significant resistance to etoposide, cisplatin and doxorubicin in hypoxia was seen in all 3 osteosarcoma cell lines, consistent with previous data showing hypoxia-induced resistance to cisplatin, doxorubicin and etoposide in a range of different tumour types. [6]–[8], [10], [11], [13], [14], [23], [25], [27], [29], [31], [35], [36], [38], [48], [52] Drug-induced apoptosis was reduced in HOS and U2OS cells exposed to cisplatin, doxorubicin and etoposide and in 791T cells exposed to cisplatin and doxorubicin, suggesting reduced apoptosis as the underlying mechanism for hypoxia-induced drug resistance. Hypoxia-induced resistance to cisplatin-induced apoptosis has been previously reported in a number of tumour cell types [25], [35], [39], [48], [52], [53] as has hypoxia-induced resistance to doxorubicin [14], [27], [35] and etoposide. [7], [10], [13], [27], [36], [53] Although 791T cells showed highly significant (p = 0.0012) resistance to etoposide in hypoxia (Figure 1) they consistently showed an equivalent degree of etoposide-induced apoptosis in normoxia and hypoxia, suggesting that other resistance mechanisms may be active in these cells.

HIF-1 is the major factor in hypoxia-induced drug resistance. Cytotoxic drug resistance in hypoxia is dependent upon functional HIF-1 in a number of different tumour cell types. [6], [8]–[11], [13], [14], [22]–[29] HIF-1 function is important for resistance to multiple cytotoxic agents including etoposide, doxorubicin and cisplatin. In a wide range of different tumour types hypoxia-induced drug resistance can be reversed by HIF-1 inhibition. [6], [8], [10], [11], [13], [14], [22], [23], [25], [27], [29] However, in the osteosarcoma cell lines HOS, U2OS and 791T stabilisation of HIF-1α in normoxia, with activation of the HIF-1 pathway, failed to induce drug resistance (Figure 3), suggesting that HIF-1 activation is not sufficient for cytotoxic drug resistance in these cells. Furthermore targeting HIF-1 in hypoxia, with several different approaches, dominant negative HIF-1, shRNAi, siRNA and the small molecule inhibitor NSC-134754, failed to reverse drug resistance in hypoxia, despite very clear evidence of functional inhibition of the HIF-1 pathway (Figures 4–6). This data suggests strongly that hypoxia-induced drug resistance is independent of HIF-1 in these osteosarcoma cells. HIF-1 independent mechanisms of drug resistance in hypoxia are under-investigated and rarely reported. However changes in apoptotic proteins can be independent of HIF-1 [8], [14], [54] and in several cell types hypoxia-induced drug resistance is only partially reversed by HIF-1 inhibition suggesting the existence of HIF-1 independent mechanisms of drug resistance. [9], [38] HIF-1 null renal proximal tubular cells remain resistant to cisplatin in hypoxia [48], and in pancreatic carcinoma cells hypoxia-induced resistance to cisplatin, doxorubicin and gemcitabine-induced apoptosis is dependent on the survival kinase Pim-1, the induction of which in hypoxia is independent of HIF-1. [35] Inhibition of PI3K, COX-2, NFκB, STAT-3 and AP-1 can reverse resistance to cytotoxic drugs in hypoxia, implying a role for these pathways in hypoxia-induced drug resistance. [12], [31], [34]–[39] However the degree to which they are dependent on functional HIF-1 is often uncertain. Previous publications reporting the HIF-1 independence of hypoxia-induced drug resistance have been contradicted subsequently by the demonstration of HIF-1 dependence, accounted for by an initial failure to adequately suppress HIF-1. Thus in HepG2 hepatoma cells initial experiments resulting in 50% reduction of HIF-1 activity suggested that resistance to etoposide-induced apoptosis in hypoxia did not depend upon HIF-1, but subsequent experiments achieving 95% reduction in HIF-1 activity showed very clear dependence on functional HIF-1. [29], [36] In HOS and 791T osteosarcoma cells functional inhibition of HIF-1, as measured by the up-regulation of protein levels of CA IX, was achieved through shRNAi (Figure 4B and 4C). In 791T cells complete loss of HIF-1α protein and functional inhibition, as measured by CA IX, was also achieved with transient transfection of siRNA (Figure 4F). In both cell lines this loss of functional HIF-1 did not reverse hypoxia-induced drug resistance. In U2OS cells transient transfection of dominant negative HIF-1α, which significantly reduced the transcription of both CA IX and GLUT-1 in hypoxia, did not alter hypoxia-induced drug resistance to all 3 drugs (Figure 5). Finally, despite inhibition of HIF-1 function by NSC-134754 in U2OS cells, hypoxia-induced resistance to all 3 cytotoxics persisted (Figure 6). Thus in these three osteosarcoma cell lines, four different methods of inhibition of HIF-1 function failed to have any effect upon hypoxia-induced drug resistance, providing strong evidence for the HIF-1 independence of this phenomenon.

Consistent with previous reports of increased activation of Akt in hypoxia, hypoxia leads to phosphorylation of Akt at serine 473 in 791T and U2OS osteosarcoma cells (Figure 7A). [43] Phosphorylation of Akt correlated with reduced levels of PTEN, also previously reported, [31], [55] and consistent with the normal regulation of this pathway. [56] Activation of the PI3K pathway is a recognised cause of cytotoxic resistance in hypoxia, protecting against both drug-induced and serum withdrawal-induced apoptosis. [12], [42], [44], [57]–[59] Mechanisms include inhibition of GSK-3 activity [43], [60], [61] and activation of NFκB. [12] Activation of the PI3K pathway may also stabilise and activate HIF-1 [62] and inactivate p53, [63] both of which are known to reduce apoptosis in hypoxia. However, although Akt is activated in hypoxia in 791T and U2OS osteosarcoma cells, inhibition of PI3K with PI-103 is not able to re-sensitise cells to cytotoxic agents, regardless of scheduling (Figure 7B), suggesting it does not contribute significantly to hypoxia-induced drug resistance in these cells. This differs from the situation in lung cancer, pancreatic cancer and phaeochromocytoma cells in which hypoxic resistance to cytotoxic-induced apoptosis is reversed by PI3K inhibition [12], [42], [44].

Modification of p53 in hypoxia is well reported [64] and its inactivation leads to a reduction in drug-induced apoptosis. [30]–[33], [48] Furthermore HIF-1 mediated inactivation of p53 in normoxia also induces chemoresistance. [37], [49] It has been suggested that, although U2OS cells have wild type p53, the pathway is non-functional because of mdm2 over-expression. [65] However, when U2OS cells are exposed to cytotoxic agents phosphorylation of p53 leads to the disassociation of p53 from mdm2, activation of down-stream targets and p53–dependent apoptosis. [66], [67] Increased p21 protein levels after cytotoxic drug exposure in our experiments imply an active downstream pathway in U2OS cells consistent with this data (Figure 8A). p53 inactivation in hypoxia (Figure 8A), may contribute to the reduced drug-induced apoptosis in hypoxia seen in U2OS osteosarcoma cells, as in HepG2 hepatoma cells (Sermius 2008). p53 inactivation in U2OS cells is not dependent on functional HIF-1 (Figure 8B and C), and this is consistent with the contribution of p53 inactivation to hypoxia-induced drug resistance. HOS cells are known to have non-functioning mutated p53, and although the p53 status of 791T cells is not described, we have not observed a p53-regulated response to DNA damaging agents. However significant hypoxia-induced drug resistance was observed in both these cell lines, although the degree of resistance was significantly greater in U2OS cells. Thus, although it may contribute to hypoxia-induced drug resistance in U2OS cells, p53 inactivation cannot be the only cause of hypoxia-induced resistance in osteosarcoma cells. Potential alternative drug resistance mechanisms including activation of NFκB [37], c-jun [31] and p-ERK 1/2 [32] have all been reported as contributing to hypoxia-induced drug resistance in cancer cells with inactive p53 pathways.

In conclusion the significant hypoxia-induced drug resistance in these three osteosarcoma cell lines suggests that hypoxia is a potential target in osteosarcoma. However the failure of HIF-1 inhibition to reverse drug resistance in hypoxia suggests that alternative approaches are needed. p53 inactivation in hypoxia may contribute to drug resistance in osteosarcoma cells with a functioning p53 pathway but cannot be the cause of drug resistance in all osteosarcoma cells. Further work is needed to identify a targetable pathway on which hypoxia-induced drug resistance in osteosarcoma is dependent.

## Methods

### Cell Culture

Osteosarcoma cell lines U2OS (ATCC), HOS (ATCC) and 791T (Paterson Institute for Cancer Research Cell Bank) were maintained in GIBCO RPMI medium with 10% FCS in 95% air and 5% CO_2_ at 37°C. All cell lines were authenticated by CRUK in July 2010 using STR profiling.

For hypoxia experiments, cells were incubated and treated in an InVivo2 Hypoxia workstation 400 (Ruskin Technology Limited) flushed with 1% O_2_, 5% CO_2_, and 94% N_2_ (subsequently referred to as hypoxia).

### Analysis of Cell Population Growth by SRB Assay

Drug response was assessed using the sulphorhodamine-B (SRB) assay. After 24 hours pre-incubation in normoxia or hypoxia cells in log phase were exposed to etoposide (Sigma-Aldrich E1383), cisplatin (Sigma-Aldrich 479306) or doxorubicin (Sigma-Aldrich D1515) for a period of 1 hour then further incubated in normoxia or hypoxia for 72 hour before processing as previously described. [68] IC_50_ values were calculated on GraphPad Prism5 software using the Hill equation and represent 50% of the drug’s maximal response.

### Protein Detection by Western Blotting

Cells were harvested for western blotting as described. [69] Primary antibodies were applied overnight in PBST or 1–5% milk in PBST: Actin (1∶1000; Sigma A4700), Akt (1∶1000; Cell Signalling 9272), CA IX (1∶1000; Bayer), Cleaved caspase-3 (1∶100; Cell Signalling 9661), GapDH (1∶2500; Sigma G9545), HIF-1α (1∶1000; BD Transduction Laboratories 610958), HIF-2α (ep190b) (1∶500; Novus Biologicals NB 100–132H), NOXA (1∶1000; Imgenex IMG-349A), p53 (1∶1000; Santa Cruz Biotechnology (Do-1) sc-126), PARP (1∶1000; Cell Signalling 9542), Phospho-Akt (Ser 473) (1∶1000; Cell Signalling (193H12) 4058), Phospho–p53 (Ser15) (1∶1000; Cell Signalling 92840), PTEN (1∶1000; BD Pharmingen 559600), PUMA (1∶1000; Sigma (bbc3-C-Terminal) P4618), WAF1 (P21) (1∶500; Oncogene OP64-100UG). Secondary antibodies were either goat anti-mouse horseradish peroxidase or goat anti-rabbit horseradish peroxidase (DAKO P0447 and P0448).

### VEGF Elisa

VEGF was determined by the Quantikine Human VEGF-A Immunoassay (R&D Systems). Cells were allowed to adhere for 24 hours, the medium replaced and flasks incubated in hypoxia or normoxia for a further 24 hours. The supernatant was then removed and analysed for VEGF-A levels in pg/ml. VEGF levels were normalised to cell number.

### Quantitative ReverseTranscription-PCR

Total RNA was isolated using the Qiagen RNeasy Kit. Reverse transcription was performed using the TaqMan Reverse Transcription Reagent Kit (Applied Biosystems) according to the manufacturer’s guidelines. Glut-1 and CA IX were amplified using the following primer sequences (shown 5′ to 3′): Glut-1 GGTTGTGCCATACTCATGACC (left primer), CAGATAGGACATCCAGGGTAGC (right primer); CA IX CCTTTGCCAGAGTTGACGAG (left primer), GCAACTGCTCATAGGCACTG (right primer) and universal probes 67 and 25 for Glut-1 and CA IX respectively (Roche). Succinate dehydrogenate complex A (SDHA), L14, L32 and beta-2-microglobulin (B2M) were housekeeper genes. RT-PCR was performed with 5ng template cDNA using TaqMan Master Mix and an ABI Prism 7900 HT sequence detection system (Applied Biosystems). Cross-threshold (C_T_) values were calculated using the 2.1 software (ABI). The 2^(−ΔΔCT)^ was calculated to represent the fold change of the target gene mRNA in hypoxia compared to normoxia [70].

### Apoptosis Assays

Cells were harvested 48 or 72 hours following drug exposure. For Annexin-V/7-AAD staining trypsinised cells were stained in 96 wells plate to identify apoptotic cells. Data were collected on BD FACSArray™ and analysed by FlowJo software. The percentage of apoptotic cells was calculated by combining all the annexin-V positive cells or 7-AAD positive cells. Western blotting was performed for cleaved caspase 3 and cleaved PARP. Morphological changes of apoptosis were assessed 48 hours after drug exposure. Cell pellets were fixed in 10% formalin (Sigma-Aldrich) and re-suspended in ProLong Gold antifade with DAPI (Molecular Probe). Apoptotic nuclear morphology was quantified using an Olympus BX51 UV fluorescence microscope, counting 3 full fields or at least 300 cells.

### Induction of HIF-1α in Normoxia using Cobalt Chloride

Cells were exposed to 24 hours cobalt chloride (50 µM 791T cells, 25 µM HOS and U2OS cells) before exposure to a range of concentrations of cisplatin or etoposide for 1 hour. An SRB assay was performed after 72 hours. Cells simultaneously plated and treated were lysed after 96 hours of cobalt chloride exposure and protein levels of HIF-1α and CA IX assessed by western blotting.

### Short Hairpin RNA Interference for HIF-1α

The HIF-1α target sequence GTCTCGAGATGCAGCCAGA [8] was incorporated into p-Silencer 2.1-U6 Hygro (Ambion (AM5760)). This plasmid was stably transfected into cells by electroporation at 1050uF, 260 V. After hygromycin selection (100 µg/ml for 791T and U2OS cells and 20 µg/ml for HOS cells), single clones were screened for HIF-1α protein expression in hypoxia. All clones were maintained in RPMI containing 10% FCS and hygromycin (100 µg/ml for 791T cells, 5 µg/ml for HOS cells and 25 µg/ml for U2OS cells).

For control the firefly luciferase target sequence CTTACGCTGAGTACTTCGA replaced the HIF-1α target sequence. Transfection and selection were as above. Single clones were transfected with the expression vector pBactin-IRES-GFP-ff-Luc using FuGENE HD transfection reagent as per the manufacturer’s instructions (Roche 04709705001). 24 hours after transfection cells were sorted on the BD FACSVantage™ SE Cell Sorter (BD Biosciences) and cells positive for GFP retained. GFP positive were incubated in medium containing streptomycin and penicillin (50units/ml, penicillin-streptomycin liquid, Invitrogen 15070-063, diluted 1∶100) for 24 hours and then subjected to a luciferase assay as per the manufacturer’s instructions (Promega E1500). Luminescence was measured on the FLUOstar OPTIMA microplate reader and normalised to cell number. Clones were selected for significant reduction in luciferase expression. HIF-1α protein levels in hypoxia in the luciferase shRNAi clones did not differ from the parental cells.

### Small Interfering RNA Interference for HIF-1α

siRNAs targeted to HIF-1α and non-targeting (NT) control siRNA were from Dharmacon SMARTpool (Thermo-Scientific L-004018-00 and D-001810-01-20). Cells were transfected with siRNA at 25 nM using the DharmaFECT 2 siRNA transfection reagent (Thermo-Scientific T-2002) according to the manufacturer’s instructions. After 24 hours siRNA was replaced by full growth medium. 6–8 hours after transfection cells were incubated in hypoxia until drug exposure 32 hours after transfection (after 24 hours in hypoxia). Simultaneously plated and transfected cells were harvested for HIF-1α and CA IX protein detection by western blotting.

### HIF-1 Inhibition by Dominant Negative HIF (DN HIF)

Both the pEF IRES-P HIF-no TAD EGFP plasmid (dominant negative HIF (DN HIF)) [6], [28] and the pEF IRES-P EGFP empty vector (EV) control were kindly donated by Dr Kaye Williams, University of Manchester. DN-HIF or EV plasmids were transiently transfected into U2OS osteosarcoma cells using FuGENE HD transfection reagent as per manufacturer’s instructions. 24 hours after transfection cells were seeded for SRB assay. Cells from the same pool were simultaneously seeded for quantification of Glut-1 and CA IX mRNA by qPCR.

### HIF-1 Inhibition by NSC-134754

Cells were treated with 20 µM NSC 134754 [71] (National Cancer Institute, Bethesda) for 24 hours in normoxia or hypoxia before exposure to cytotoxic for 1 hour. The concentration of 20 µM NSC-134754 was maintained throughout. Identical plates without NSC 134754 treatment were used as controls. After 72 hours an SRB assay was performed. Protein levels of HIF-1 and CA IX were determined in simultaneously plated cells by western blotting.

### Activation of Akt and PI3K Inhibition

Protein levels of total Akt, Akt pS473, and PTEN were assessed after 48 hours incubation in normoxia and hypoxia by western blotting. After a 24 hour incubation period in normoxia or hypoxia cells were treated with 1 µM PI-103 (Calbiochem 528100) followed by cytotoxic for 1 hour. After 72 hours an SRB assay was performed. The concentration of 1 µM PI-103 was maintained throughout. Cells simultaneously plated and treated with and without PI-103 were harvested at the end of the experiment (after 96 hours of PI-103 treatment) for western blotting.

### p53 Activation in Response to Cytotoxic Treatment and the Influence of HIF-1

24 hours after exposure to SRB IC_50_ doses of cytotoxic for 1 hour lysates were assessed for total p53, p53 pS15, p21^(WAF1)^, PUMA and NOXA by western blotting. U2OS cells were transiently transfected with the DN HIF or EV plasmids. 24 hours after transfection cells were pre-incubated in normoxia or hypoxia for 24 hours then exposed to 1 µM etoposide. 24 hours later protein levels were assessed by western blotting. Simultaneously transfected and plated cells were maintained in normoxia or hypoxia and harvested at 24 hours for qPCR for CA IX and Glut-1 expression.

### Statistics

Statistical significance of differences was assessed using 2-tailed student’s *t* test or 2-way ANOVA, a *p* value of less than 0.05 considered significant. Experiments show the average of 3 independent experiments unless otherwise stated and western blots are representative of 3 independent experiments. Error bars indicate SEM.

## References

[1] Stiller CA (2007) International patterns of cancer incidence in adolescents. Cancer Treat Rev 33: 631–645. 17329031 10.1016/j.ctrv.2007.01.001

[2] Gatta G, Corazziari I, Magnani C, Peris-Bonet R, Roazzi P, et al (2003) Childhood cancer survival in Europe. Ann Oncol 14 Suppl 5v119–127. 14684502 10.1093/annonc/mdg755

[3] Link MP, Goorin AM, Miser AW, Green AA, Pratt CB, et al (1986) The effect of adjuvant chemotherapy on relapse-free survival in patients with osteosarcoma of the extremity. N Engl J Med 314: 1600–1606. 3520317 10.1056/NEJM198606193142502

[4] Harting MT, Blakely ML, Jaffe N, Cox CS Jr, Hayes-Jordan A, et al (2006) Long-term survival after aggressive resection of pulmonary metastases among children and adolescents with osteosarcoma. J Pediatr Surg 41: 194–199. 16410132 10.1016/j.jpedsurg.2005.10.089

[5] Meyers PA, Heller G, Healey JH, Huvos A, Applewhite A, et al (1993) Osteogenic sarcoma with clinically detectable metastasis at initial presentation. J Clin Oncol 11: 449–453. 8445419 10.1200/JCO.1993.11.3.449

[6] Brown LM, Cowen RL, Debray C, Eustace A, Erler JT, et al (2006) Reversing hypoxic cell chemoresistance in vitro using genetic and small molecule approaches targeting hypoxia inducible factor-1. Mol Pharmacol 69: 411–418. 16254058 10.1124/mol.105.015743

[7] Dai S, Huang ML, Hsu CY, Chao KS (2003) Inhibition of hypoxia inducible factor 1alpha causes oxygen-independent cytotoxicity and induces p53 independent apoptosis in glioblastoma cells. Int J Radiat Oncol Biol Phys 55: 1027–1036. 12605983 10.1016/s0360-3016(02)04507-8

[8] Erler JT, Cawthorne CJ, Williams KJ, Koritzinsky M, Wouters BG, et al (2004) Hypoxia-mediated down-regulation of Bid and Bax in tumors occurs via hypoxia-inducible factor 1-dependent and -independent mechanisms and contributes to drug resistance. Mol Cell Biol 24: 2875–2889. 15024076 10.1128/MCB.24.7.2875-2889.2004 PMC371100

[9] Ravizza R, Molteni R, Gariboldi MB, Marras E, Perletti G, et al (2009) Effect of HIF-1 modulation on the response of two- and three-dimensional cultures of human colon cancer cells to 5-fluorouracil. Eur J Cancer 45: 890–898. 19171477 10.1016/j.ejca.2008.12.021

[10] Song X, Liu X, Chi W, Liu Y, Wei L, et al (2006) Hypoxia-induced resistance to cisplatin and doxorubicin in non-small cell lung cancer is inhibited by silencing of HIF-1alpha gene. Cancer Chemother Pharmacol 58: 776–784. 16532342 10.1007/s00280-006-0224-7

[11] Sullivan R, Pare GC, Frederiksen LJ, Semenza GL, Graham CH (2008) Hypoxia-induced resistance to anticancer drugs is associated with decreased senescence and requires hypoxia-inducible factor-1 activity. Mol Cancer Ther 7: 1961–1973. 18645006 10.1158/1535-7163.MCT-08-0198

[12] Yokoi K, Fidler IJ (2004) Hypoxia increases resistance of human pancreatic cancer cells to apoptosis induced by gemcitabine. Clin Cancer Res 10: 2299–2306. 15073105 10.1158/1078-0432.ccr-03-0488

[13] Hussein D, Estlin EJ, Dive C, Makin GW (2006) Chronic hypoxia promotes hypoxia-inducible factor-1alpha-dependent resistance to etoposide and vincristine in neuroblastoma cells. Mol Cancer Ther 5: 2241–2250. 16985058 10.1158/1535-7163.MCT-06-0145

[14] Kilic M, Kasperczyk H, Fulda S, Debatin KM (2007) Role of hypoxia inducible factor-1 alpha in modulation of apoptosis resistance. Oncogene 26: 2027–2038. 17043658 10.1038/sj.onc.1210008

[15] Kaya M, Wada T, Akatsuka T, Kawaguchi S, Nagoya S, et al (2000) Vascular endothelial growth factor expression in untreated osteosarcoma is predictive of pulmonary metastasis and poor prognosis. Clin Cancer Res 6: 572–577. 10690541

[16] Mizobuchi H, Garcia-Castellano JM, Philip S, Healey JH, Gorlick R (2008) Hypoxia markers in human osteosarcoma: an exploratory study. Clin Orthop Relat Res 466: 2052–2059. 18528739 10.1007/s11999-008-0328-y PMC2493019

[17] Yang QC, Zeng BF, Dong Y, Shi ZM, Jiang ZM, et al (2007) Overexpression of hypoxia-inducible factor-1alpha in human osteosarcoma: correlation with clinicopathological parameters and survival outcome. Jpn J Clin Oncol 37: 127–134. 17237146 10.1093/jjco/hyl137

[18] Chen WL, Feng HJ, Li HG (2008) Expression and significance of hypoxemia-inducible factor-1alpha in osteosarcoma of the jaws. Oral Surg Oral Med Oral Pathol Oral Radiol Endod 106: 254–257. 18554951 10.1016/j.tripleo.2008.01.029

[19] Ema M, Taya S, Yokotani N, Sogawa K, Matsuda Y, et al (1997) A novel bHLH-PAS factor with close sequence similarity to hypoxia-inducible factor 1alpha regulates the VEGF expression and is potentially involved in lung and vascular development. Proc Natl Acad Sci U S A 94: 4273–4278. 9113979 10.1073/pnas.94.9.4273 PMC20712

[20] Wang GL, Jiang BH, Rue EA, Semenza GL (1995) Hypoxia-inducible factor 1 is a basic-helix-loop-helix-PAS heterodimer regulated by cellular O2 tension. Proc Natl Acad Sci U S A 92: 5510–5514. 7539918 10.1073/pnas.92.12.5510 PMC41725

[21] Maxwell PH, Wiesener MS, Chang GW, Clifford SC, Vaux EC, et al (1999) The tumour suppressor protein VHL targets hypoxia-inducible factors for oxygen-dependent proteolysis. Nature 399: 271–275. 10353251 10.1038/20459

[22] Comerford KM, Wallace TJ, Karhausen J, Louis NA, Montalto MC, et al (2002) Hypoxia-inducible factor-1-dependent regulation of the multidrug resistance (MDR1) gene. Cancer Res 62: 3387–3394. 12067980

[23] Sullivan R, Graham CH (2009) Hypoxia prevents etoposide-induced DNA damage in cancer cells through a mechanism involving hypoxia-inducible factor 1. Mol Cancer Ther 8: 1702–1713. 19509259 10.1158/1535-7163.MCT-08-1090

[24] Liu XW, Su Y, Zhu H, Cao J, Ding WJ, et al (2010) HIF-1alpha-dependent autophagy protects HeLa cells from fenretinide (4-HPR)-induced apoptosis in hypoxia. Pharmacol Res 62: 416–425. 20637870 10.1016/j.phrs.2010.07.002

[25] Hao J, Song X, Song B, Liu Y, Wei L, et al (2008) Effects of lentivirus-mediated HIF-1alpha knockdown on hypoxia-related cisplatin resistance and their dependence on p53 status in fibrosarcoma cells. Cancer Gene Ther 15: 449–455. 18421307 10.1038/cgt.2008.4

[26] Huang L, Ao Q, Zhang Q, Yang X, Xing H, et al (2010) Hypoxia induced paclitaxel resistance in human ovarian cancers via hypoxia-inducible factor 1alpha. J Cancer Res Clin Oncol 136: 447–456. 19760195 10.1007/s00432-009-0675-4 PMC11828325

[27] Liu L, Ning X, Sun L, Zhang H, Shi Y, et al (2008) Hypoxia-inducible factor-1 alpha contributes to hypoxia-induced chemoresistance in gastric cancer. Cancer Sci 99: 121–128. 17953712 10.1111/j.1349-7006.2007.00643.x PMC11158535

[28] Roberts DL, Williams KJ, Cowen RL, Barathova M, Eustace AJ, et al (2009) Contribution of HIF-1 and drug penetrance to oxaliplatin resistance in hypoxic colorectal cancer cells. Br J Cancer 101: 1290–1297. 19755992 10.1038/sj.bjc.6605311 PMC2768443

[29] Sermeus A, Cosse JP, Crespin M, Mainfroid V, de Longueville F, et al (2008) Hypoxia induces protection against etoposide-induced apoptosis: molecular profiling of changes in gene expression and transcription factor activity. Mol Cancer 7: 27. 18366759 10.1186/1476-4598-7-27 PMC2330149

[30] Achison M, Hupp TR (2003) Hypoxia attenuates the p53 response to cellular damage. Oncogene 22: 3431–3440. 12776195 10.1038/sj.onc.1206434

[31] Cosse JP, Ronvaux M, Ninane N, Raes MJ, Michiels C (2009) Hypoxia-induced decrease in p53 protein level and increase in c-jun DNA binding activity results in cancer cell resistance to etoposide. Neoplasia 11: 976–986. 19794957 10.1593/neo.09632 PMC2745664

[32] Wang D, Zhu Q, Zhang X, Zhang L, He Q, et al (2010) Hypoxia promotes etoposide (VP-16) resistance in neuroblastoma CHP126 cells. Pharmazie 65: 51–56. 20187579

[33] Zhang L, Hill RP (2004) Hypoxia enhances metastatic efficiency by up-regulating Mdm2 in KHT cells and increasing resistance to apoptosis. Cancer Res 64: 4180–4189. 15205329 10.1158/0008-5472.CAN-03-3038

[34] Boller YC, Brandes LM, Russell RL, Lin ZP, Patierno SR, et al (2000) Prostaglandin A1 inhibits stress-induced NF-kappaB activation and reverses resistance to topoisomerase II inhibitors. Oncol Res 12: 383–395. 11697817 10.3727/096504001108747846

[35] Chen J, Kobayashi M, Darmanin S, Qiao Y, Gully C, et al (2009) Pim-1 plays a pivotal role in hypoxia-induced chemoresistance. Oncogene 28: 2581–2592. 19483729 10.1038/onc.2009.124 PMC3358117

[36] Piret JP, Cosse JP, Ninane N, Raes M, Michiels C (2006) Hypoxia protects HepG2 cells against etoposide-induced apoptosis via a HIF-1-independent pathway. Exp Cell Res 312: 2908–2920. 16844113 10.1016/j.yexcr.2006.05.018

[37] Rohwer N, Dame C, Haugstetter A, Wiedenmann B, Detjen K, et al (2010) Hypoxia-inducible factor 1alpha determines gastric cancer chemosensitivity via modulation of p53 and NF-kappaB. PLoS One 5: e12038. 20706634 10.1371/journal.pone.0012038 PMC2919384

[38] Schnitzer SE, Schmid T, Zhou J, Brune B (2006) Hypoxia and HIF-1alpha protect A549 cells from drug-induced apoptosis. Cell Death Differ 13: 1611–1613. 16456580 10.1038/sj.cdd.4401864

[39] Selvendiran K, Bratasz A, Kuppusamy ML, Tazi MF, Rivera BK, et al (2009) Hypoxia induces chemoresistance in ovarian cancer cells by activation of signal transducer and activator of transcription 3. Int J Cancer 125: 2198–2204. 19623660 10.1002/ijc.24601 PMC2893222

[40] Park SY, Billiar TR, Seol DW (2002) Hypoxia inhibition of apoptosis induced by tumor necrosis factor-related apoptosis-inducing ligand (TRAIL). Biochem Biophys Res Commun 291: 150–153. 11829475 10.1006/bbrc.2002.6421

[41] Carroll VA, Ashcroft M (2006) Role of hypoxia-inducible factor (HIF)-1alpha versus HIF-2alpha in the regulation of HIF target genes in response to hypoxia, insulin-like growth factor-I, or loss of von Hippel-Lindau function: implications for targeting the HIF pathway. Cancer Res 66: 6264–6270. 16778202 10.1158/0008-5472.CAN-05-2519

[42] Alvarez-Tejado M, Naranjo-Suarez S, Jimenez C, Carrera AC, Landazuri MO, et al (2001) Hypoxia induces the activation of the phosphatidylinositol 3-kinase/Akt cell survival pathway in PC12 cells: protective role in apoptosis. J Biol Chem 276: 22368–22374. 11294857 10.1074/jbc.M011688200

[43] Chen EY, Mazure NM, Cooper JA, Giaccia AJ (2001) Hypoxia activates a platelet-derived growth factor receptor/phosphatidylinositol 3-kinase/Akt pathway that results in glycogen synthase kinase-3 inactivation. Cancer Res 61: 2429–2433. 11289110

[44] Lee SM, Lee CT, Kim YW, Han SK, Shim YS, et al (2006) Hypoxia confers protection against apoptosis via PI3K/Akt and ERK pathways in lung cancer cells. Cancer Lett 242: 231–238. 16427189 10.1016/j.canlet.2005.11.001

[45] Damia G, Filiberti L, Vikhanskaya F, Carrassa L, Taya Y, et al (2001) Cisplatinum and taxol induce different patterns of p53 phosphorylation. Neoplasia 3: 10–16. 11326311 10.1038/sj.neo.7900122 PMC1505020

[46] Lowe SW, Ruley HE, Jacks T, Housman DE (1993) p53-dependent apoptosis modulates the cytotoxicity of anticancer agents. Cell 74: 957–967. 8402885 10.1016/0092-8674(93)90719-7

[47] Graeber TG, Osmanian C, Jacks T, Housman DE, Koch CJ, et al (1996) Hypoxia-mediated selection of cells with diminished apoptotic potential in solid tumours. Nature 379: 88–91. 8538748 10.1038/379088a0

[48] Wang J, Biju MP, Wang MH, Haase VH, Dong Z (2006) Cytoprotective effects of hypoxia against cisplatin-induced tubular cell apoptosis: involvement of mitochondrial inhibition and p53 suppression. J Am Soc Nephrol 17: 1875–1885. 16762987 10.1681/ASN.2005121371

[49] Roberts AM, Watson IR, Evans AJ, Foster DA, Irwin MS, et al (2009) Suppression of hypoxia-inducible factor 2alpha restores p53 activity via Hdm2 and reverses chemoresistance of renal carcinoma cells. Cancer Res 69: 9056–9064. 19920202 10.1158/0008-5472.CAN-09-1770 PMC2789194

[50] Kalra R, Jones AM, Kirk J, Adams GE, Stratford IJ (1993) The effect of hypoxia on acquired drug resistance and response to epidermal growth factor in Chinese hamster lung fibroblasts and human breast-cancer cells in vitro. Int J Cancer 54: 650–655. 8514457 10.1002/ijc.2910540421

[51] Teicher BA, Lazo JS, Sartorelli AC (1981) Classification of antineoplastic agents by their selective toxicities toward oxygenated and hypoxic tumor cells. Cancer Res 41: 73–81. 7448778

[52] Song J, Qu Z, Guo X, Zhao Q, Zhao X, et al (2009) Hypoxia-induced autophagy contributes to the chemoresistance of hepatocellular carcinoma cells. Autophagy 5: 1131–1144. 19786832 10.4161/auto.5.8.9996

[53] Koch S, Mayer F, Honecker F, Schittenhelm M, Bokemeyer C (2003) Efficacy of cytotoxic agents used in the treatment of testicular germ cell tumours under normoxic and hypoxic conditions in vitro. Br J Cancer 89: 2133–2139. 14647149 10.1038/sj.bjc.6601375 PMC2376846

[54] Dong Z, Venkatachalam MA, Wang J, Patel Y, Saikumar P, et al (2001) Up-regulation of apoptosis inhibitory protein IAP-2 by hypoxia. Hif-1-independent mechanisms. J Biol Chem 276: 18702–18709. 11278985 10.1074/jbc.M011774200 PMC2854569

[55] Graziani I, Eliasz S, De Marco MA, Chen Y, Pass HI, et al (2008) Opposite effects of Notch-1 and Notch-2 on mesothelioma cell survival under hypoxia are exerted through the Akt pathway. Cancer Res 68: 9678–9685. 19047145 10.1158/0008-5472.CAN-08-0969

[56] Vivanco I, Sawyers CL (2002) The phosphatidylinositol 3-Kinase AKT pathway in human cancer. Nat Rev Cancer 2: 489–501. 12094235 10.1038/nrc839

[57] Clark AS, West K, Streicher S, Dennis PA (2002) Constitutive and inducible Akt activity promotes resistance to chemotherapy, trastuzumab, or tamoxifen in breast cancer cells. Mol Cancer Ther 1: 707–717. 12479367

[58] Hovelmann S, Beckers TL, Schmidt M (2004) Molecular alterations in apoptotic pathways after PKB/Akt-mediated chemoresistance in NCI H460 cells. Br J Cancer 90: 2370–2377. 15150572 10.1038/sj.bjc.6601876 PMC2409515

[59] Opel D, Westhoff MA, Bender A, Braun V, Debatin KM, et al (2008) Phosphatidylinositol 3-kinase inhibition broadly sensitizes glioblastoma cells to death receptor- and drug-induced apoptosis. Cancer Res 68: 6271–6280. 18676851 10.1158/0008-5472.CAN-07-6769

[60] Dai T, Zheng H, Fu GS (2008) Hypoxia confers protection against apoptosis via the PI3K/Akt pathway in endothelial progenitor cells. Acta Pharmacol Sin 29: 1425–1431. 19026161 10.1111/j.1745-7254.2008.00904.x

[61] Risbud MV, Fertala J, Vresilovic EJ, Albert TJ, Shapiro IM (2005) Nucleus pulposus cells upregulate PI3K/Akt and MEK/ERK signaling pathways under hypoxic conditions and resist apoptosis induced by serum withdrawal. Spine (Phila Pa 1976) 30: 882–889. 15834331 10.1097/01.brs.0000159096.11248.6d

[62] Blancher C, Moore JW, Robertson N, Harris AL (2001) Effects of ras and von Hippel-Lindau (VHL) gene mutations on hypoxia-inducible factor (HIF)-1alpha, HIF-2alpha, and vascular endothelial growth factor expression and their regulation by the phosphatidylinositol 3′-kinase/Akt signaling pathway. Cancer Res 61: 7349–7355. 11585776

[63] Sabbatini P, McCormick F (1999) Phosphoinositide 3-OH kinase (PI3K) and PKB/Akt delay the onset of p53-mediated, transcriptionally dependent apoptosis. J Biol Chem 274: 24263–24269. 10446202 10.1074/jbc.274.34.24263

[64] Hammond EM, Giaccia AJ (2005) The role of p53 in hypoxia-induced apoptosis. Biochem Biophys Res Commun 331: 718–725. 15865928 10.1016/j.bbrc.2005.03.154

[65] Florenes VA, Maelandsmo GM, Forus A, Andreassen A, Myklebost O, et al (1994) MDM2 gene amplification and transcript levels in human sarcomas: relationship to TP53 gene status. J Natl Cancer Inst 86: 1297–1302. 8064888 10.1093/jnci/86.17.1297

[66] Jackson MW, Agarwal MK, Agarwal ML, Agarwal A, Stanhope-Baker P, et al (2004) Limited role of N-terminal phosphoserine residues in the activation of transcription by p53. Oncogene 23: 4477–4487. 15064747 10.1038/sj.onc.1207575

[67] Yuan XW, Zhu XF, Huang XF, Sheng PY, He AS, et al (2007) Interferon-alpha enhances sensitivity of human osteosarcoma U2OS cells to doxorubicin by p53-dependent apoptosis. Acta Pharmacol Sin 28: 1835–1841. 17959036 10.1111/j.1745-7254.2007.00662.x

[68] Vichai V, Kirtikara K (2006) Sulforhodamine B colorimetric assay for cytotoxicity screening. Nat Protoc 1: 1112–1116. 17406391 10.1038/nprot.2006.179

[69] Klymenko T, Brandenburg M, Morrow C, Dive C, Makin G (2011) The novel Bcl-2 inhibitor ABT-737 is more effective in hypoxia and is able to reverse hypoxia-induced drug resistance in neuroblastoma cells. Mol Cancer Ther 10: 2373–2383. 22006676 10.1158/1535-7163.MCT-11-0326 PMC3242074

[70] Livak KJ, Schmittgen TD (2001) Analysis of relative gene expression data using real-time quantitative PCR and the 2(-Delta Delta C(T)) Method. Methods 25: 402–408. 11846609 10.1006/meth.2001.1262

[71] Chau NM, Rogers P, Aherne W, Carroll V, Collins I, et al (2005) Identification of novel small molecule inhibitors of hypoxia-inducible factor-1 that differentially block hypoxia-inducible factor-1 activity and hypoxia-inducible factor-1alpha induction in response to hypoxic stress and growth factors. Cancer Res 65: 4918–4928. 15930314 10.1158/0008-5472.CAN-04-4453

